# PCR Detection of *Bartonella* spp. and *Borreliella* spp. DNA in Dry Blood Spot Samples from Human Patients

**DOI:** 10.3390/pathogens13090727

**Published:** 2024-08-28

**Authors:** Kerry L. Clark, Shirley Hartman

**Affiliations:** 1 Department of Public Health, University of North Florida, 1 UNF Drive, Jacksonville, FL 32224, USA; 2 Mandarin Wellness Center, Jacksonville, FL 32257, USA

**Keywords:** *Bartonella*, *Borrelia burgdorferi*, PCR, dry blood spots

## Abstract

Lyme disease is the most commonly reported vector-borne disease in the United States. *Bartonella* constitute an additional zoonotic pathogen whose public health impact and diversity continue to emerge. Rapid, sensitive, and specific detection of these and other vector-borne pathogens remains challenging, especially for patients with persistent infections. This report describes an approach for DNA extraction and PCR testing for the detection of *Bartonella* spp. and *Borreliella* spp. from dry blood spot (DBS) specimens from human patients. The present study included extraction of DNA and PCR testing of DBS samples from 105 patients with poorly defined, chronic symptoms labeled as Lyme-Like Syndromic Illness (LLSI). *Bartonella* spp. DNA was detected in 20/105 (19%) and *Borreliella* spp. DNA was detected in 41/105 (39%) patients with LLSI. Neither group of organisms was detected in DBS samples from 42 healthy control subjects. *Bartonella* spp. 16S–23S rRNA internal transcribed spacer sequences were highly similar to ones previously identified in yellow flies, lone star ticks, a human patient from Florida, mosquitoes in Europe, or *B. apihabitans* and *choladocola* strains from honeybees. These human strains may represent new genetic strains or groups of human pathogenic species of *Bartonella*. The 41 *Borreliella* spp. *flaB* gene sequences obtained from human patients suggested the presence of four different species, including *B. burgdorferi*, *B. americana, B. andersonii*, and *B. bissettiae*/*carolinensis*-like strains. These results suggest that specific aspects of the DBS DNA extraction and PCR approach enabled the detection of *Bartonella* spp. and *Borreliella* spp. DNA from very small amounts of human whole blood from some patients, including specimens stored on filter paper for 17 years.

## 1. Introduction

During the past 30 years, *Bartonella* spp. have been increasingly recognized as causes of human infections worldwide and responsible for diverse clinical manifestations [1,2,3]. *Bartonella* spp. infect a wide variety of mammalian host species and can be transmitted by various blood-feeding arthropods, including fleas, body lice, sand flies, bat flies, and mites; evidence is growing that ticks are also vectors of some *Bartonella* spp. [1,2]. At least 12 *Bartonella* spp. are associated with human infections, with *B. henselae* being most commonly identified [4]. Despite their widespread occurrence and the breadth of species associated with human infections, *Bartonella* spp. infection (bartonellosis) is not a reportable disease in the USA; this lack of surveillance likely hampers awareness among clinicians and the general public, disease diagnosis, and accurate estimation of true disease frequency.

*Bartonella* spp. use a variety of mechanisms to evade the host immune system and maintain persistent infections [5,6]. Culture isolation of *Bartonella* spp. is laborious and challenging, due in part to transient bacteremia, while antibody testing lacks both sensitivity and specificity [4]. A combined enrichment culture and PCR approach improved the ability to identify *Bartonella* spp. in clinical samples [7] and continues to be optimized [8,9], but may still fail to identify some *Bartonella* spp. or strains.

Lyme disease (LD) is caused by bacterial spirochetes in the *Borreliella* genus [10], formerly referred to as the *Borrelia burgdorferi* sensu lato complex, and is transmitted to humans mostly by *Ixodes* spp. ticks [11]. Lyme disease is the most commonly reported vector-borne disease in the United States. More than 62,000 cases of LD were reported to the Centers for Disease Control and Prevention in 2022 following a change in the surveillance case definition allowing reporting of cases by laboratory evidence alone for high-incidence areas [12]. Confirmation of cases in areas of low incidence still requires objective clinical data in addition to laboratory evidence [12]. Evaluations of surveillance and survey data estimate that the true number of new infections may be well over 400,000 cases each year [13].

Lyme disease can lead to multisystem manifestations affecting skin, joints, the nervous system, or the heart [14]. It is believed that most cases recover following antibiotic treatment, but some go on to experience chronic health problems that can persist for years [11]. Debate continues over the cause of persistent symptoms following infection and standard antibiotic treatment, including permanent damage from initial infection, autoimmune responses, or ongoing infection [15]. Persistent infection following antibiotic monotherapy has been demonstrated in animal models [16] and in some cases may be documented with clinical proteomics [17].

Although LD in the USA is most frequently caused by *B. burgdorferi*, evidence has shown human infection with other *Borreliella* spp., including *B. mayonii*, *B. bissettiae*, *B. americana*, and *B. andersonii* [18,19,20,21,22]. However, studies evaluating the ability of commonly used clinical laboratory tests to detect infections with strains from species other than *B. burgdorferi* are lacking. Furthermore, it is widely acknowledged that the standard two-tier antibody testing approach for LD has poor sensitivity and other limitations in detecting both early and later-stage infections [23].

Clearly, there is a need for more sensitive detection methods for both *Bartonella* and Lyme *Borreliella* spp. infections in human patients. The purpose of this study was to investigate the cause of chronic Lyme-like syndromic illness (LLSI) among human patients, and to evaluate a dry blood spot (DBS) polymerase chain reaction (PCR) testing approach for *Bartonella* and *Borreliella* spp. as potential causes of the patients’ clinical syndromes. This report describes methods for and results of testing over 100 patients with LLSI from Florida and a few other states for DNA evidence of *Bartonella* spp. and *Borreliella* spp. using very small volumes of whole blood collected and stored as DBSs. The methods described below enabled the detection of divergent strains of as-yet-uncharacterized *Bartonella* spp. and four species of *Borreliella* among study patients, many of whom had been chronically ill for years.

## 2. Materials and Methods

### 2.1. Human Patient and Control Subjects

Beginning in 2003, the University of North Florida Public Health Research Laboratory (UNFPHRL) began receiving human blood specimens for research testing from patients experiencing multi-systemic symptoms consistent with acute or persistent Lyme disease (UNF IRB approvals #06-140 and #468310, covering the period from 2003 to present). We refer to this constellation of symptoms as LLSI. The patients, who at the time of study enrollment lacked confirmed laboratory evidence for Lyme disease, were evaluated by one of the authors (SH) at her clinical practice for signs and symptoms consistent with Lyme disease. Beginning in 2007, finger prick blood samples (dry blood spots, DBS) were collected onto filter paper from some patients for long-term blood storage for research testing. A questionnaire was completed for each patient to obtain basic demographic data, travel history prior to onset of signs/symptoms, tick exposure, and clinical data. Most patients were not tested at clinical laboratories for *Bartonella*, but many were tested for Lyme antibodies via IgM/IgG Western Blot. Some of them were tested previously by other clinicians prior to this study, and data on some patients’ test results were not available to the authors.

As part of the present study, we also obtained DBS samples from 42 healthy control patient samples obtained from UNF employees and students. The control subjects also completed a questionnaire that included an extensive checklist of signs and symptoms related to Lyme disease as a means of screening out subjects with possible undiagnosed arthropod-borne disease or autoimmune diseases. These patient and control DBS specimens were used for evaluating the DNA extraction and testing methods described below.

### 2.2. Sample Collection and PCR Testing

Whole blood from study subjects was obtained via finger sticks using BD Microtainer^®^ contact-activated lancets (Becton, Dickinson and Company, Franklin Lakes, NJ, USA). Blood was blotted onto FTA^TM^ cards (Qiagen, Germantown, MD, USA), Whatman^TM^ 3MM paper (Cytiva Life Sciences, Marlborough, MA, USA), or 903 Five Spot Blood Card paper (EBF-Inc., Mauldin, SC, USA) as separate drops of ~10–15 μL. For some specimens, 3MM paper was cut into 5-mm-wide strips, and blood drops were added until the sampling portion of the strip was saturated. Blood spots were allowed to air dry completely before storage. Some were stored in individual plastic centrifuge tubes, while others were stored in individual small coin envelopes, all of which were placed in separate zip-closure biohazard bags and kept in a laboratory refrigerator at 0–4 °C, some for up to 17 years prior to testing during this study.

DNA extractions from DBS samples included between one and two 5 × 5 mm squares (from some 3MM paper strips) or 6-mm-diameter round blood-soaked filter paper punches from various paper types. Each square paper piece or round punch was estimated to contain ~10–12.5 μL of blood. These samples were either cut from filter paper strips or sheets with stainless steel scissors or punched from paper with a standard metal single hole paper punch. Forceps, scissors, or punch used to handle these samples were soaked first in 5% sodium hypochlorite (bleach) for 1 min, then in 2.5% sodium hypochlorite for 1 min, then rinsed in 70% ethanol, and flamed with an alcohol burner between each individual sample. Additionally, 6 dry scissor cuts or punches were made in a separate clean piece of 3MM filter paper between samples as an additional measure to prevent any carryover contamination. Clean filter paper samples were included in extractions as negative controls for PCR testing. For DNA extraction, DBSs were added directly to lysis solution and treated as tissue specimens. All patient specimens were extracted with a salting out procedure with reagents from the MasterPure Kit (Biosearch Technologies, Petaluma, CA, USA), with some modifications of the manufacturer’s protocols, as described below.

DBS paper samples were added to 530 µL of 1× Tissue and Cell Lysis buffer plus 10 µL of 20 mg/mL proteinase K in 2 mL low-retention microcentrifuge tubes. Lysis was carried out for 3 h at 55 °C. Tubes were then centrifuged for 3 min at 21,000× *g*, and all liquid was transferred to clean tubes, leaving behind the paper pieces. Approximately 500 μL of liquid was transferred, as some remains absorbed by the paper. Samples were then chilled at −20 °C for 8 min. A volume of 300 µL of MPC Protein Precipitation reagent was added, and samples were vortexed thoroughly (~15 s). Tubes were chilled again at −20 °C for 8 min and centrifuged for 10 min at 16,000× *g* at 20 °C in a refrigerated centrifuge to pellet protein. The liquid was transferred to clean tubes, avoiding carryover of any protein precipitate. Then, 5 µL of polyacryl carrier (MRC, Cincinnati, OH, USA) was added and samples were mixed thoroughly. A volume of 800 µL of 100% isopropanol was added, and tubes were inverted 50 times and bump vortexed for 5 s. Samples were stored overnight at 0–4 °C to precipitate DNA. The next day, tubes were centrifuged at 21,000× *g* for 30 min at 20 °C to pellet DNA. All liquid was removed from each tube using a pipet and discarded. Each DNA pellet and the inside of each tube was washed with 1 mL of 80% ethanol by gently inverting and rotating each tube 5 times by hand. Tubes were then centrifuged at 21,000× *g* for 10 min. As much liquid as possible was removed with a pipet, and pellets were air-dried completely at room temperature with the caps of the tubes open. DNA pellets were rehydrated with 60 µL of tris-EDTA buffer (pH 8.0).

Extracted DNA was tested by PCR for a fragment of the human beta-globin gene to identify potential inhibition and to demonstrate amplifiable DNA. Primers PC04 (5′-CAA-CTT-CAT-CCA-CGT-TCA-CC-3′) and GH20 (5′-GAA-GAG-CCA-AGG-ACA-GGT-AC-3′) amplify a 268-bp fragment [24]. These and all other reactions utilized GoTaqGreen PCR master Mix (Promega, Madison, WI, USA) in a total volume of 25 μL and 5 μL of extracted DNA. All PCR tests began with a denaturation step at 94 °C, 2 min. Primer concentration for all PCRs was 0.25 μM. Beta-globin gene PCRs included 30 cycles of amplification with denaturation at 94 °C, 30 s, primer annealing at 52 °C, 30 s, and extension at 72 °C, 30 s.

DNA extracts were then tested by PCR for the presence of *Bartonella* spp. 16S–23S rRNA intergenic spacer (ITS) and *Borreliella* spp. *flaB* gene DNA. The PCR primers and protocols were previously described [25,26]. However, in the present study, we made slight modifications to those protocols. *Bartonella* spp. ITS primers used to initially screen patients were 325S (5′-CCT-CAG-ATG-ATG-ATC-CCA-AGC-CTT-CTG-GCG-3′) and 1100AS (5′-GAA-CCG-ACG-ACC-CCC-TGC-TTG-CAA-AGC-A-3′). These primers amplify variable-sized products from different *Bartonella* species, ranging from approximately 275-bp to more than 900-bp. The *Borreliella* spp. *flaB* PCR primers designed by our lab were 313F (5′-GCA-GAC-AGA-GGT-TCT-ATA-CAA-ATT-GAA-ATA-GAG-C-3′), and 551R (5′-GCT-TCA-TCT-TGG-TTT-GCT-CCA-ACA-TGA-ACT-C-3′), which amplify a 239-bp fragment. For *Bartonella* spp. ITS, initial denaturation was followed by 55 cycles of 92 °C, 30 s, primer annealing at 66 °C, 30 s, and extension at 72 °C, 30 s. The *Borreliella* spp. *flaB* PCRs consisted of a total of 55 cycles, beginning with a touchdown phase of 10 cycles beginning with an annealing temperature of 65 °C and dropping one degree per cycle, followed by 45 additional cycles of 92 °C, 30 s; 60 °C, 30 s; and 72 °C, 30 s. Negative control PCR samples containing nuclease-free water in place of a template were included along with DNA extraction controls in each round of PCR testing at a rate of one control for every six experimental samples. PCR products were electrophoresed in 2% agarose gels containing ethidium bromide and visualized under UV light to identify potential positive results.

### 2.3. DNA Sequence Analysis

All PCR products of the expected range for positive results were sequenced with both primers used for PCR using standard Sanger sequencing at Eurofins Genomics (Louisville, KY, USA). Forward and reverse primer sequences from each sample were aligned using Clustal Omega [27] and compared via BLAST [28] search with sequences in the GenBank database. Representative *Bartonella* spp. ITS DNA sequences obtained during the study were assigned GenBank numbers PP955084-PP955093. Representative *Borreliella* spp. *flaB* DNA sequences were assigned GenBank numbers PP942627-PP942650. A multiple sequence alignment of investigator-derived *Bartonella* ITS sequences combined with reference species sequences was conducted with Clustal Omega [27]. Following a model test in MEGA [29,30], a phylogenetic comparison was made using the Maximum Likelihood method and Jukes Cantor model [31] in MEGA. A bootstrap consensus tree was inferred using 1000 replicates [32].

### 2.4. Data and Statistical Analyses

Concentration and A260/A280 absorbance ratios of DNA extracted from patient and control DBSs were determined with a Nanodrop Lite spectrophotometer (ThermoFisher Scientific, Waltham, MA, USA). Means and standard deviations of subject group age and gender, DNA concentration, and A260/A280 ratios were calculated with Microsoft^®^ Excel, version 16.88. Statistical comparisons of subject age groups, gender, extracted DNA concentration and A260/A280 ratios, symptom prevalence, and serological results between PCR-positive and PCR-negative patients were conducted with the EngineRoom (MoreSteam^®^, Powell, OH, USA) software package. All statistical comparisons were made with an alpha (significance) level of 0.05.

## 3. Results

### 3.1. Human Patient and Control Subjects

DBS samples were available for testing from 105 patient subjects sampled between 2007 and 2024. The number of patients sampled, shown in parentheses, by year were: 2007 (30), 2011 (12), 2012 (34), 2013 (1), 2015 (8), 2016 (7), 2019 (3), 2022 (1), 2023 (7), 2024 (2). These samples provided 113 total DNA extracts because two samples were tested from eight patients. Thus, samples were denoted as Hs-DBS-1 through Hs-DBS-113. Most (94%) of the patients were residents of Florida. The number of patients tested from other states included one each from Georgia, Kentucky, North Carolina, New York, Texas, and Virginia. For comparison, DBS samples were collected from 42 controls (all from Florida). 

Approximately 60% of the patients reported a history of tick bite, compared to 10% of the control subjects. Around 10% of the patients had previously been diagnosed with Lyme disease or another tickborne disease by a different clinician. Only 18% reported a history of erythema migrans rash. The most common complaints of patients were fatigue, headache, muscle or joint pain, unspecified neurological symptoms, and unrestful sleep. Twenty-five percent of the patients had experienced symptoms for more than a year, and over 20% reported being ill for 4–5 years or longer.

The age range of patients was 6–87 years, and the mean and median ages of patients were 42.8 years and 45 years, respectively. The age range of controls was 19–47 years, and the mean and median ages of controls were 23 years and 21 years, respectively. The mean age of patients was significantly greater than for controls (*p* < 0.05), likely because most controls were university students. There were significantly more female patients than males (61.9% vs. 38.1%, *p <* 0.05), and significantly more female controls than males (78.6% vs. 21.4%, *p* < 0.05).

### 3.2. DNA Concentration and Quality

All subject DBS DNA extracts successfully amplified the targeted fragment of the human beta-globin gene, demonstrating no apparent PCR inhibition. The DNA concentration of DBS extracts from control subjects ranged from 22.1 to 35.7 ng/µL, with a mean concentration of 27.1 ng/µL and standard deviation (SD) of 4.85. The range of A260/A280 ratios of control samples was 1.68–1.75, with a mean of 1.72, SD = 0.0254. The DNA concentration of patient DBS extracts ranged from 14.4 to 69.5 ng/µL, with a mean concentration of 32.67 ng/µL, and SD = 12.49. The range of A260/A280 ratios for patient samples was 1.52–1.92, with a mean of 1.69 and SD = 0.0757. The mean DNA concentration for patient samples was significantly greater (*p* = 0.0006), and the mean A260/A280 ratio was significantly lower (*p* = 0.0015) than for controls. The DNA concentration range for PCR-positive (*Bartonella* spp. or *Borreliella* spp.) patients (see below) was 20.6–63.1 ng/µL, with mean of 36.56 ng/µL and SD = 15.34, and the range was 14.4–69.5 ng/µL, with mean of 31.58 ng/µL and SD = 10.86 for PCR-negative patients. The mean DNA concentration did not differ significantly between PCR-positive and PCR-negative patients (*p* = 0.055). However, the mean A260/A280 ratio of 1.67 (range 1.52–1.78, SD = 0.0688) for PCR-positive patient samples was significantly lower (*p* = 0.0393) than for PCR-negative patients (mean 1.7, range 1.53–1.92, SD = 0.0770).

### 3.3. PCR Testing and DNA Sequence Analysis

All control subject DBS DNA extracts, negative control filter paper extracts, and PCR reagent negative control samples tested negative for both *Bartonella* spp. and *Borreliella* spp. throughout the study, demonstrating a lack of DNA artifact contamination. *Bartonella* spp. ITS DNA was detected in 20/105 = 19% of patients. The number of positive patients from different states included 18 from Florida, and 1 each from Kentucky and New York. Lyme *Borreliella* spp. *flaB* DNA was detected in 41/105 = 39% of patients. The number of positive patients by state was 36 from Florida, and 1 each from Georgia, Kentucky, North Carolina, New York, and Texas. A total of 48 (45.7%) patients tested positive for either *Bartonella* spp. or *Borreliella* spp. DNA. Approximately 10% (11/105) of patients tested positive for both pathogen groups. At least one pathogen group was detected in samples collected nearly every year. The age of the DBS sample did not appear to negatively affect detection; for example, at least one pathogen group was detected in 11/30 (37%) samples from 2007 that were ~17 years old when tested, 7/12 (58%) samples from 2011, and 15 of 34 (44%) samples from 2012. The proportion of *Bartonella* spp. or *Borreliella* spp. positive patients was not significantly different among males (37.5%) vs. females (50.77%) (*p* = 0.185).

In comparing the proportion of PCR positive (for either pathogen group) vs. negative patients, there was no significant difference in the proportions reporting EM rash (0% vs. 6%), fatigue (40% vs. 22%), pain in the arms or legs (20% vs. 11%), neurological symptoms (30% vs. 17%), or unrestful sleep (20% vs. 17%). PCR-positive patients did experience significantly higher rates than PCR-negative patients of chills and fever (30% vs. 11%; *p* = 0.0154), headache (40% vs. 11%; *p* = 0.0005), muscle or joint pain (50% vs. 22%; *p* = 0.0037), paralysis (20% vs. 6%; *p* = 0.0158), and meningitis (20% vs. 0%; *p* = 0.0003).

Lyme Western Blot antibody test results from clinical labs were available for 75 patients. Lyme antibody enzyme immunoassay screen tests were either not conducted or results from tests conducted by other clinicians prior to this study were not available to the authors. None of the patients were IgG positive, which requires the detection of at least 5 of 10 antibody bands considered in the standard test panels. The most commonly detected IgG antibody was p41, present in 56% of tested patients. Only 10% of tested patients had positive IgM results, based on the criteria of two of three IgM bands present (including p23, p39, p41) for positive results. The most commonly detected IgM bands were p23 (34%) and p41 (29%). Patients with IgM-positive WB results were not more likely than IgM-negative patients to test PCR-positive for *Borreliella* spp. (*p* > 0.05).

### 3.4. Borreliella *spp.* Detected

The majority (32/41 = 78%) of *Borreliella* spp. detected was *B. burgorferi*-like strains; the other positives included *B. americana*-like strains (5/41 = 12%), *B. andersonii*-like strains (3/41 = 7%), and one *B. bissettiae*/*carolinensis*-like strain that could not be differentiated more specifically with the small *flaB* fragment sequence obtained with our test.

### 3.5. Bartonella *spp.* DNA Sequence Analysis

All *Bartonella* spp. ITS sequences detected in human patients were 97–100% similar to recently detected strains from lone star ticks from Virginia, USA [33], mosquitoes from Europe [34], honeybees from China [35], or lone star ticks, yellow flies, and a human patient from FL, USA [26]. None of the *Bartonella* strains were similar to the most commonly reported species infecting humans or other vertebrates (for example, *B. henselae*, *B. quintana*, or others).

Because of some DNA mismatch and ambiguous bases in the 5′ region of the human patient sample ITS sequences, a new ITS forward primer was developed that matched nearly 100% of all the human sequences and aforementioned closely related sequences in Genbank. This 30-nt primer (5′-CCC-AAG-CCT-TCT-GGC-GAC-CTG-ACA-GAT-TGT-3′) was designed to match the melting temperature of the published 28-nt reverse primer 1100AS, and therefore used the same PCR annealing temperature of 66 °C. With this new forward primer, PCR experiments with human DBS extracts showed improved specificity (for example, much less primer–dimer) and better sensitivity for the strains detected, with a lower amplification cycle number of 50. However, mismatch in the 3′ end of the new forward primer with ITS sequences from other *Bartonella* spp. probably renders it unable to detect other *Bartonella* genetic groups.

**Figure 1:**
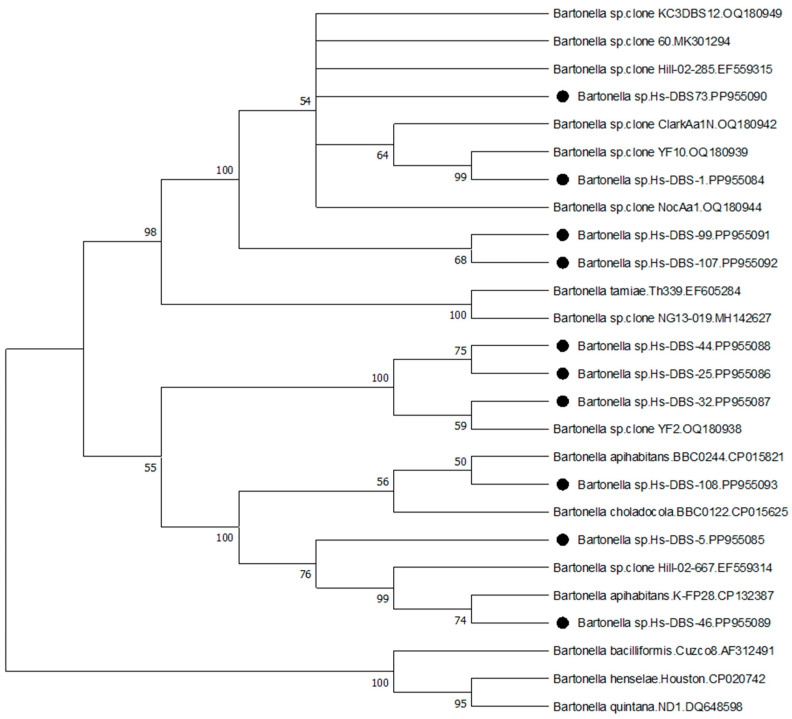
Evolutionary analysis by the maximum likelihood method. The evolutionary history was inferred by using the maximum likelihood method and Jukes–Cantor model [31]. The bootstrap consensus tree inferred from 1000 replicates [32] is taken to represent the evolutionary history of the taxa analyzed. Branches corresponding to partitions reproduced in less than 50% bootstrap replicates are collapsed. The percentage of replicate trees in which the associated taxa clustered together in the bootstrap test are shown next to the branches [32]. Initial tree(s) for the heuristic search were obtained automatically by applying Neighbor-Join and BioNJ algorithms to a matrix of pairwise distances estimated using the Jukes–Cantor model, and then selecting the topology with superior log likelihood value. The rate variation model allowed for some sites to be evolutionarily invariable ([+*I*], 53.39% sites). This analysis involved 26 nucleotide sequences. All positions with less than 95% site coverage were eliminated, i.e., fewer than 5% alignment gaps, missing data, and ambiguous bases were allowed at any position (partial deletion option). There was a total of 254 positions in the final dataset. Evolutionary analyses were conducted in MEGA11 [30]. *Bartonella* ITS sequences derived from human patients in this study are denoted with black circles.

The size of ITS sequences obtained from human patients with the new ITS primer combination ranged from 259-bp to 285-bp. (see Supplementary Materials). This region was used for comparison and phylogenetic analysis of representative human strain sequences with reference sequences in Genbank. Figure 1 shows the bootstrap consensus tree obtained by the Maximum Likelihood method. Our ITS sequences from human DBS samples were grouped into three clusters (Figure 1 and Supplementary Materials). Of the sequences included in the analysis, three patient strains, Hs-DBS-5, 46, and 108, were between 97 and 100% similar to, and cluster with, *B. apihabitans* and *B. choladochola* strains from honeybees [35], and clone Hill-02-667 from a lone star tick in Virginia, USA [33]. Three strains, Hs-DBS-25, 32, and 44, were 98–99% similar to, and cluster with, clone YF2 obtained from a yellow fly in Florida [26]. The other human DBS strains were 99% similar to, and loosely cluster with, clone 60 from a mosquito in Europe [34], a different lone star tick from Virginia (clone Hill-02-285) [33], and strains from lone star ticks, yellow flies, and a previously described human patient in Florida [26].

## 4. Discussion

It is widely acknowledged that a need exists for more sensitive and specific diagnostic tests for multiple arthropod-borne pathogens, including *Bartonella* spp. [4] and Lyme *Borreliella* species [36]. This is especially true in the case of chronic and antibiotic-resistant infections. Molecular methods show promise for improved sensitivity and specificity, and depending upon design, the capability to detect previously undetected strains/species of pathogens [4,25,33,36,37,38]. Dry blood spot samples have been used as a template for pathogen identification by molecular methods for some years [39,40]. The UNFPHRL has used them for over 20 years, and experimented with methods to improve their use, for the detection of multiple tickborne pathogens from wild vertebrates [41,42,43] and a human patient in a previous report [26]. 

The salting out DNA extraction method utilizing reagents from the Masterpure kit has been shown capable of high DNA recovery and the ability to recover small DNA fragments [39,44,45,46,47,48]. In comparison testing in our lab for the past 20 years, this extraction method has demonstrated superior DNA recovery and resulting PCR detection rates for tickborne pathogens compared to other methods, such as spin column kits from multiple manufacturers. In addition, our PCR testing with the primers and protocols used in this study enabled the detection of *Bartonella* spp. and *Borreliella* spp. DNA from chronically ill patients using only single reaction amplification. Potential reasons for the observed sensitivity and specificity are that the primers amplify relatively small DNA fragments of the ITS region of detected strains of *Bartonella* spp. and *Borreliella* spp. *flaB*, the length of the primers (~30-nt long), and relatively high annealing temperatures used. Additionally, the combination of this DNA extraction method and these PCR assays may be capable of detecting cell-free circulating DNA from low levels of whole-cell bacteria in the bloodstream.

Advantages of this DNA extraction and PCR approach using DBS samples include the following: ease of DBS sample collection; non-invasive procedure with low risk of adverse events for patients; low biosafety risk when handling DBS samples; stability of samples and DNA integrity over time; high DNA recovery from very small amounts of template; highly sensitive PCR tests capable of detecting low concentration of target DNA; and ability of the tests to potentially differentiate *Bartonella* spp. genetic groups and *Borreliella* spp. Disadvantages of our testing approach include: sample handling for the DNA extraction is somewhat labor intensive and time-consuming (requiring an overnight DNA precipitation step for improved sensitivity); requirement of more hands-on skill than automated and some other manual extraction methods; three sets of microtubes are needed per sample; the current approach requires DNA sequencing for confirmation of positive results; the improved *Bartonella* spp. ITS PCR probably only detects the newly identified genetic groups; and the *Borreliella* spp. *flaB* PCR might not be able to differentiate all species. Despite the advantages and disadvantages, further refinement and optimization of the presented protocols is likely possible. For example, combining the presented DNA extraction method and PCR primers with nested PCR primers for each respective group of pathogens might provide even greater detection sensitivity; however, nested PCR could increase the risk of DNA amplicon contamination and thus false-positive PCR results.

We identified diverse *Bartonella* strains and ITS strain variants in human patients residing in different states that clustered with, or close to, recently recognized genetic groups, such as *B. tamiae* from human patients in Thailand [49], uncharacterized strains from lone star ticks from Virginia, USA [33], mosquitoes in Europe [34], *B. apihabitans* and *B. choladocola* from honeybees [35], and lone star ticks, yellow flies, and a human patient in Florida USA [26]. Results of the ITS sequence alignment and phylogenetic analysis suggest the possibility of three genetic groups or possibly three distinct *Bartonella* species associated with these human infections. In the present study, one patient from Virginia had symptom onset immediately after multiple yellow jacket stings, raising questions about possible transmission from stinging insects. Another patient from Kentucky with a similar strain had a history of both bumblebee stings and tick bites. Other patients had documented tick bites, some with confirmed lone star tick bites, and others with unidentified tick species. Despite using a previously published *Bartonella* spp. ITS PCR for initial screening, we did not detect any strains of the more commonly reported species, such as *B. henselae*, *B. quintana*, or others. Our results thus question whether these recognized species are the most common cause of *Bartonella* spp. infection in the southern USA. Future studies need to focus on efforts to culture these newly recognized strains to allow for comprehensive characterization, possible vector transmission experiments, and other studies.

We identified a relatively high (39%) prevalence of *Borreliella* spp. in human patients from southern states with persistent symptoms of LLSI. The species distribution detected in this study’s patients was very similar to that determined in a previous study by the UNFPHRL [22]. Here, we documented four different species, from most to least prevalent, *B. burgdorferi*, *B. americana*, *B. andersonii*, and *B. bissettiae/carolinensis*. These results support our previous findings [20,21,22], showing that multiple *Borreliella* species infect humans in the southern U.S. It is not known how well the standard two-tier antibody testing for LD can identify infection with southern strains of *B. burgdorferi*, or the other species detected. However, despite the fact that almost all patients in the present study were chronically ill, none were Lyme WB IgG positive. Additionally, Lyme WB IgM positivity was only 10% and did not correlate with PCR-positive results for *Borreliella* spp. This finding supports concerns regarding the sensitivity of current Lyme serological tests [36]. 

Almost all patients included in this study were chronically ill, and more than 20% of them for 4–5 years or longer. *Bartonella* spp. and *Borreliella* spp. are known to cause persistent infections in their natural hosts [5,50], and therefore, they likely operate similarly in humans. In the present study, nearly 50% of the study patients tested positive for sequence-confirmed *Bartonella* spp. or Lyme *Borreliella* spp. infection, and 10% tested positive for both groups. Due to the extremely small blood samples utilized in our testing, we believe that our detection prevalence is a conservative estimate of true infection in the study population. If so, this suggests that *Bartonella* and Lyme *Borreliella* infection are not rare in Florida and perhaps other southern states. Previous studies have described finding *Bartonella* or *Borreliella* in patients from southern states with similar clinical syndromes [3,20,21,22,26,51]. Although our results do not definitively prove that the study patients’ illnesses were caused by the detected *Bartonella* or *Borreliella* strains, they do suggest a plausible cause. Additional studies of human patients with similar clinical syndromes are warranted.

## Data Availability

The raw data supporting the conclusions of this article will be made available by the authors on request.

## Supplementary Materials

The following supporting information can be downloaded at: https://www.mdpi.com/article/10.3390/pathogens13090727/s1, Table S1: Clustal alignment file of Hs-DBS-*Bartonella* ITS sequences.
