# Supplementary material for: PCR Detection of Bartonella spp. and Borreliella spp. DNA in Dry Blood Spot Samples from Human Patients
Source: Pathogens. 2024 Aug 28;13(9):727. doi: 10.3390/pathogens13090727 (PMC11435347; doi:10.3390/pathogens13090727)
Supplement: Supplementary file 1 [file pathogens-13-00727-s001.zip › pathogens-3105769-supplementary.pdf]

CLUSTAL O(1.2.4) multiple sequence alignment of Bartonella spp. 16S-23S rRNA ITS sequences

```
Hs-DBS1.PP955084        CCCAAGCCTTCTGGCGACCTGACGGATTGTAAGGTGCGTTGCCGGT-AGGTTTGCCGGTA   59
Hs-DBS73.PP955090       CCCAAGCCTTCTGGCGACCTGACAGATTGTAAGGTGCGTTGCCGGT-AGGTTTGCCGGTA   59
Hs-DBS99.PP955091       CCCAAGCCTTCTGGCGACCTGACAGATTGTAAGGTGCGTTGCCGGT-AGGTTTGCCGGTA   59
Hs-DBS107.PP955092      CCCAAGCCTTCTGGCGACCTGACAGATTGTAAGGTGCGTTGCCGGT-AGGTTTGCCGGTA   59
Hs-DBS44.PP955088       CCCAAGCCTTCTGGCGACCTGACAGATTGTAAGGTGCGTAGCCGGTTAGGTTTGCCGGTG   60
Hs-DBS25.PP955086       CCCAAGCCTTCTGGCGACCTGACAGATTGTAAGGTGCGTAGCCGGTTAGGTTTGCCGGTG   60
Hs-DBS32.PP955087       CCCAAGCCTTCTGGCGACCTGACAGATTGTAAGGTGCGTAGCCGGTTAGGTTTGCCGGTG   60
Hs-DBS108.PP955093      CCCAAGCCTTCTGGCGACCTGACAGATTGTAAGGTGCGGTGCTGGTGAGGTTTGCCGGCA   60
Hs-DBS5.PP955085        CCCAAGCCTTCTGGCGACCTGACAGATTGTAAGGTGCGGTGCTGGTGAGGTTTGCCGGCA   60
Hs-DBS46.PP955089       CCCAAGCCTTCTGGCGACCTGACAGATTGTAAGGTGCGGTGCTGGTGAGGTTTGCCGGCA   60
                        *********************** **************  ** *** ***********  

Hs-DBS1.PP955084        GACGTAAGTTTGCCGAGGGCTTGTAGCTCAGTTGGTTAGAGCGCGCGCTTGATAAGCGTG   119
Hs-DBS73.PP955090       GACGTAAGTTTGCCGAGGGCTTGTAGCTCAGTTGGTTAGAGCGCGCGCTTGATAAGCGTG   119
Hs-DBS99.PP955091       GACGTAAGTTTGCCGAGGGCTTGTAGCTCAGTTGGTTAGAGCGCGCGCTTGATAAGCGTG   119
Hs-DBS107.PP955092      GACGTAAGTTTGCCGAGGGCTTGTAGCTCAGTTGGTTAGAGCGCGCGCTTGATAAGCGTG   119
Hs-DBS44.PP955088       AATGTGAATTTGCCGAGGGCTTGTAGCTCAGTTGGTTAGAGCGCGCGCTCGATAAGCGTG   120
Hs-DBS25.PP955086       AATGTGAATTTGCCGAGGGCTTGTAGCTCAGTTGGTTAGAGCGCGCGCTTGATAAGCGTG   120
Hs-DBS32.PP955087       AATGTGAATTTGCCGAGGGCTTGTAGCTCAGTTGGTTAGAGCGCGCGCTTGATAAGCGTG   120
Hs-DBS108.PP955093      --GCGGAAACTGCCGAGGGCTTGTAGCTCAGTTGGTTAGAGCGCGCGCTTGATAAGCGTG   118
Hs-DBS5.PP955085        --GCGGAAACTGCCGAGGGCTTGTAGCTCAGTTGGTTAGAGCGCGCGCTTGATAAGCGTG   118
Hs-DBS46.PP955089       --GCGGAAACTGCCGAGGGCTTGTAGCTCAGTTGGTTAGAGCGCGCGCTTGATAAGCGTG   118
                              *   *************************************** **********

Hs-DBS1.PP955084        AGGTCGGAGGTTCAAGTCCTCCCAGGCCCACCAATATTTGCGCTCATAATGTATTGGCTT   179
Hs-DBS73.PP955090       AGGTCGGAGGTTCAAGTCCTCCCAGGCCCACCAATATTTGCGCTCATAATGTATTGGCTT   179
Hs-DBS99.PP955091       AGGTCGGAGGTTCAAGTCCTCCCAGGCCCACCAATATTTGCGCTCATAATGTATTGGCTT   179
Hs-DBS107.PP955092      AGGTCGGAGGTTCAAGTCCTCCCAGGCCCACCAATATTTGCGCTCATAATGTATTGGCTT   179
Hs-DBS44.PP955088       AGGTCGGAGGTTCAAGTCCTCCCAGGCCCACCATATTGCGCTCACTGTTTGGAGTTAGCT   180
Hs-DBS25.PP955086       AGGTCGGAGGTTCAAGTCCTCCCAGGCCCACCATATTGCGCTCACTGTTTGGAGTTAGCA   180
Hs-DBS32.PP955087       AGGTCGGAGGTTCAAGTCCTCCCAGGCCCACCATATTGCGCTCACTGTTTGGAGTTAGC-   179
```

```
Hs-DBS108.PP955093      AGGTCGGAGGTTCAAGTCCTCCCAGGCCCACCAAGAATTGCGCTTCACTAAAATGG----   174
Hs-DBS5.PP955085        AGGTCGGAGGTTCAAGTCCTCCCAGGCCCACCAAGAATTGCGCTTCGCCGA-ATGG----   173
Hs-DBS46.PP955089       AGGTCGGAGGTTCAAGTCCTCCCAGGCCCACCAAGAATTGCGCTTCGCCGA-ATGG----   173
                        *********************************      ** *         *

Hs-DBS1.PP955084        TTAGCC-------------------TTTGTGTTGTTGAGATAAAGTTTAGGGGCCATAGC   220
Hs-DBS73.PP955090       TTAGCC-------------------TTTGTGTTGTTGAGATAGAGTTTAGGGGCCATAGC   220
Hs-DBS99.PP955091       TCAGCC-------------------TTTGTGTTGTTGAGATAAAGTTTAGGGGCCATAGC   220
Hs-DBS107.PP955092      TCAGCC-------------------TTTGTGTTGTTGAGATAAAGTTTAGGGGCCATAGC   220
Hs-DBS44.PP955088       --ATAAGATTGCTATGGACTCAAATGGATGTGAGTATATAAAAGTATTAGGGGCCATAGC   238
Hs-DBS25.PP955086       ATATAAGATTGCTATGAACTCAAATGGATGTGAGTATATAAAAGTATTAGGGGCCATAGC   240
Hs-DBS32.PP955087       ATATAAGATTGCTATGAACTCAAATGGATGTGAGTATATAAAAGTATTAGGGGCCATAGC   239
Hs-DBS108.PP955093      ------------------AGACATTTGGTGAGAGGGCATGGAAGTCTAAGGGGCCATAGC   216
Hs-DBS5.PP955085        ------------------AGACATTTGGTAGGAAGCGAAAAAAGTTTAAGGGGCCATAGC   215
Hs-DBS46.PP955089       ------------------AGACATTTGGTGAGAGGCA-TGAAAGTCTAAGGGGCCATAGC   214
                                                                 *    * ************

Hs-DBS1.PP955084        TCAGCTGGGAGAGCACCTGCTTTGCAAGCAGGGGGCCGTCGGTTC    265
Hs-DBS73.PP955090       TCAGCTGGGAGAGCACCTGCTTTGCAAGCAGGGGGTCGTCGGTTC    265
Hs-DBS99.PP955091       TCAGCTGGGAGAGCACCTGCTTTGCAAGCAGGGGGTCGTCGGTTC    265
Hs-DBS107.PP955092      TCAGCTGGGAGAGCACCTGCTTTGCAAGCAGGGGGTCGTCGGTTC    265
Hs-DBS44.PP955088       TCAGCTGGGAGAGCACCTGCTTTGCAAGCAGGGGGTCGTCGGTTC    283
Hs-DBS25.PP955086       TCAGCTGGGAGAGCACCTGCTTTGCAAGCAGGGGGTCGTCGGTTC    285
Hs-DBS32.PP955087       TCAGCTGGGAGAGCACCTGCTTTGCAAGCAGGGGGCCGTCGGTTC    284
Hs-DBS108.PP955093      TCAGCTGGGAGAGCACCTGCTTTGCAAGCAGGGGGTCGTCGGTTC    261
Hs-DBS5.PP955085        TCAGCTGGGAGAGCACCTGCTTTGCAAGCAGGGGGTCGTCGGTTC    260
Hs-DBS46.PP955089       TCAGCTGGGAGAGCACCTGCTTTGCAAGCAGGGGGTCGTCGGTTC    259
                        *********************************** *********
```
